# Case of Peritonitis

**Published:** 1844-07-13

**Authors:** James C. Branden


### THE MEDICAL EXAMINER,

### And Record of Medical Science.

Vol. VII.] PHILADELPHIA, SATURDAY, JULY 13, 1844. [No. 14.

### CASE OF PERITONITIS

*In the Lying-in Department of the Philadelphia Hospital—terminating fatally.*

BY JAMES C. BRANDEN, M. D.

M. B., aged 17 years—of a delicate conformation—complexion simply florid, not sanguine—hair scarcely dark—eyes dark—was delivered of a living female child, weighing three pounds and a quarter, on Wednesday, May 30th. On the day previous she was seized with a sore throat, which was attended with considerable febrile action. The morning after her accouchment, the fever was quite intense; the anterior portion of the thorax and the abdomen were covered with a scarlet rash: under the use of neutral mixture the fever subsided and the rash disappeared in the course of 48 hours. The affection of the throat persisted a few days longer, but was finally relieved, after the administration of 8 grains of blue mass, the application of leeches to the angle of the jaw, and the use of a gargle of creosote and chlorinated soda.

*Sunday, June 9th.* She has been up and about the ward for several days past, and apparently doing as well as any of the women who were confined a day or two previous to her. To-day, however, she is complaining of *griping* pains in the abdomen. She was ordered to take a dose of laudanum, and to repeat it, with two tea-spoonfuls of oil, if not relieved. Her appetite has been very indifferent since her confinement.

*Monday, June 10th.* Early this morning she was complaining of pain in the abdomen, and the nurse gave her the oil and laudanum, as directed. Said she was quite well now; that "her pain had entirely gone."

*Tuesday, June 11th, 9 A. M.* Was much surprised upon approaching her bed this morning; I had never seen any person's countenance so materially altered in so short a time. Yesterday it was calm and placid and her cheeks suffused by a rosy blush common to her in health; to-day her face is *very* pale, eyes sunken and staring, and surrounded by a dark halo, nose sharpened, lips dry and retracted tightly on the gums, exposing the teeth, and the countenance, taken as a whole, offering an expression of anxiety and suffering *peculiarly striking*, and impossible to be described. Learned that she was up all day yesterday, and was quite cheerful; that during the night she was attacked with diarrhœa, was "off and on the stool all night," the precise number of evacuations not known, the dejections of a dark color and copious. Decubitus upon the back, with the lower extremities stretched out. Tongue slightly red around the edges—moist, and covered with a white fur in the centre posteriorly. Skin moderately hot and dry. Pulse rapid, quick, and small—136 in a minute. Abdomen painful on pressure, over almost its entire surface. Ordered 30 drops of laudanum by injection immediately; to have a hop poultice applied constantly over the abdomen, and to take every two hours a pill containing $\frac{1}{3}$ of a grain of opium, calomel $\frac{1}{2}$ grain, and ipecac. $\frac{1}{3}$ of a grain.

6 *P. M.* Says she has not near so much pain, "that it does not hurt her to turn over, like it did in the morning." Tenderness much diminished—seems to be confined chiefly to right iliac and lumbar regions. Tongue still moist around the edges, but has become dry and coated brown in the centre. Considerable thirst—complete anorexia; skin still hot and dry; pulse the same as in the morning; some tympanites; lochia continue, and white in colour. Has not had much milk, but it has not diminished since her illness. The injection administered in the morning was retained, and her bowels have not been opened since. Hop poultice feels very grateful—it and the pills continued. Ice water for a drink.

*Wednesday, June 12th, 9 A. M.* Slept very little—quite restless during the night; was seized with vomiting, of a greenish yellow fluid, early in the morning. Continued, if possible, worse than yesterday; eyes more anxious and sunken—the dark halo increased in size and intensity. Face pale—lips still retracted, dry and scaly—no sordes on them or teeth. Tongue dry—the brown coat extending nearly to the edges. Skin hot and dry. Pulse quick and small—between 130 and 140. Pain in abdomen extending over umbilical and hypogastric regions. Tympanites increased. Lies on her back, legs stretched out. No alvine evacuation. Mental faculties perfect—quick. Cannot be prevailed on to partake of any nutriment. Hop poultice constantly applied. Opium $\frac{1}{3}$ grain, calomel $\frac{1}{2}$ grain every two hours—ipecac. left out on account of the irritability of the stomach. Three drops of oil of turpentine, every two hours, alternately with pills.

2 *P. M.* Saw her, with Dr. Conway. Patient very much alarmed—afraid to go to sleep on account of shortness of breath. Respiration thoracic, 30 in a minute. Tongue more moist than in the morning. No change in other symptoms, except pulse, which was somewhat fluttering, on account of which we gave her 5 grains of carbonate of ammonia every two hours, with brandy punch occasionally. Turpentine and assafœtida by enema.—Pills continued.

After returning to my room, deemed it wise to send for Professor Huston, who arrived about 5 o'clock, P. M. He thought it better not to stimulate her, for fear of aggravating the local inflamation, and, therefore, the ammonia and brandy punch were stopped. He also recommended 1 grain of blue mass with 10 drops of oil of turpentine in mucilage, every 2 hours, and cloths wet with oil of turpentine to be applied to the abdomen, and hop poultice placed over them. Her diet arrow-root, chicken water, &c.

9 *P. M.* She has become more composed—disposed to sleep. Ate some arrow-root, but none of the chicken water. No marked change in any of her symptoms.

*Thursday, June 13th.* Slept some in the fore part of the night; towards morning was again attacked with emesis, vomiting every time she took her medicine. Decubitus on back, limbs stretched out. Disposition to dose—no stupor; intelligence perfect. Countenance as yesterday—awfully depressed. Some moisture on forehead—skin of trunk and extremities hot and dry. Pulse small and fluttering—near 140. Respiration thoracic, 32. Tongue dry and coated brown. No evacuation from the bowels. Turpentine has vesicated the abdomen. Between 10 and 11 o'clock Dr. Huston saw her—he now thought that the only chance of saving her was by rapid mercurialisation: and with this view he ordered the blistered surface to be dressed with mercurial ointment, and 5 grains of calomel to be given every four hours by the mouth. To allay the irritability of the stomach 1½ drops of nitric acid in a table-spoonful of camphor water was given every half hour. Injections of turpentine and lac assafœtida were administered without relieving the tympanites.

9 *P. M.* Decubitus upon the back, with the lower *extremities drawn up* (first time). The emesis has been allayed. Tongue a little moist around the edges. Skin of face and extremities moist, but warm. Pulse upwards of 150, small and fluttering. Takes no nourishment. Milk still secreted. Lochia sanguineous. No evacuation from bowels.

*Friday, June 14th*, 6 *A. M.* Skin moist—both extremities keep warm. Pulse scarcely perceptible. Patient evidently moribund.

She expired about 10 o'clock, retaining her mental faculties and power of speech to the last moment.

*Autopsy—twenty-four hours after death.*

A crucial incision was made through the abdominal parietes and the flaps turned over. Considerable adipose tissue between the integuments and the muscles. The peritoneum lining the abdominal muscles was slightly adherent to the omentum majus; the latter was spread out beautifully over the intestines, being of a deep red colour, and studded here and there with flakes of lymph; the lower portion of it was somewhat darker in colour than that above, and was pretty firmly attached to the anterior margin of the pelvis. This adhesion being loosened, those between the omentum and intestines gave way easily, and the former was turned up, and found to be nearly uniformly half an inch thick. The surfaces of the liver, stomach and spleen were covered with lymph, the folds of the intestines intimately agglutinated. The pelvic viscera, (bladder, uterus, ovaries, broad ligaments and rectum) all thickly coated with lymph. The pelvic cavity and right and left lumbar regions contained a few ounces of a cream-coloured fluid, with floculi of lymph floating in it. The peritoneal surface of most of the convolutions of the intestines presented a uniform bright red injection, and especially in the ilium, vessels of a bright red colour could be traced running from the mesentery to the intestines, and there anastamosing by a series of beautiful arcades. Commencing at the duodenum, the intestines, to the junction of the ileum with the colon, were carefully drawn between the thumb and forefinger, and in no part of them could any thing like an enlarged gland be felt. Then commencing at its junction with the caput coli, several feet of the ileum were laid open; its mucous membrane presented a smooth, *uninjected*, velvety appearance, and not a gland of Peyer or Brunner was elevated. The uterus and its appendages were now removed from the pelvis: upon dividing the fallopian tubes a small quantity of a purulent fluid exuded from their cavities. The ovaries were laid open in their long diameter, the cut surfaces offering a white texture, with here and there a jet of blood. The uterus was contracted down to a size twice as large as in the unimpregnated state; it also was laid open in its long diameter, its muscular texture was pale and firm, its cavity entirely empty, its lining membrane healthy; a circular spot about the size of a dollar at the fundus near the orifice of the left fallopian tube showed where the placenta had been attached.

